# Spectral-Efficient End-to-End Beamforming for 6G XL-MIMO: Synergizing Channel Sensing and Spatial–Frequency Sparsity with Deep Learning

**DOI:** 10.3390/s26072012

**Published:** 2026-03-24

**Authors:** Ya Wen, Xiaoping Zeng, Xin Xie

**Affiliations:** 1 School of Electronics and Internet of Things, Chongqing Polytechnic University of Electronic Technology, Chongqing 401331, China; wenya@cquet.edu.cn; 2 School of Microelectronics and Communication Engineering, Chongqing University, Chongqing 401331, China; 3 School of Automation, Chongqing University of Posts and Telecommunications, Chongqing 400065, China; xiexin@cqupt.edu.cn

**Keywords:** 6G, extremely large-scale MIMO (XL-MIMO), channel sensing, near-field communications, deep learning, end-to-end learning, sparse representation

## Abstract

Extremely Large-Scale Multiple-Input Multiple-Output (XL-MIMO) is positioned as a transformative technology for sixth-generation (6G) networks, effectively turning base stations into high-resolution sensing and communication hubs. However, the practical deployment of XL-MIMO is hindered by the “curse of dimensionality,” specifically the prohibitive overhead associated with Channel State Information (CSI) sensing and feedback, alongside the computational latency of massive antenna arrays. To resolve the conflict between high-resolution sensing requirements and limited bandwidth resources, this paper proposes a novel two-stage beamforming architecture that synergizes physics-aware dimensionality reduction with deep learning. First, by exploiting the inherent sparsity of XL-MIMO channels in the angle-delay domain, we design a Spatial–Frequency Concentration Block (SFCB). This module functions as a hard-attention sensing mechanism, performing efficient source-end dimensionality reduction on raw CSI at the User Equipment (UE) via precise feature extraction and adaptive energy truncation. Second, we develop a highly adaptable Direct Integrated Precoding Network (DIP-I). Departing from the conventional “sense-reconstruct-then-precode” paradigm, DIP-I learns end-to-end mapping to directly regress the optimal precoding matrix at the Base Station (BS). Comprehensive simulations utilizing the COST 2100 and QuaDRiGa hybrid channel models demonstrate that, under a massive 512-antenna configuration, the proposed framework achieves exceptional beamforming gain. Furthermore, it significantly reduces sensing data overhead and inference latency, offering a superior trade-off between spectral efficiency and hardware resource consumption for future 6G sensing-communication integrated systems.

## 1. Introduction

As the global research community pivots toward the sixth-generation (6G) mobile communication era, the boundary between sensing and communication is becoming increasingly blurred. The demand for ubiquitous connectivity, holographic communication, and the tactile internet has driven antenna technologies toward unprecedented scales [1]. Extremely Large-Scale Multiple-Input Multiple-Output (XL-MIMO) has emerged as a pivotal enabler to meet these rigorous demands. By deploying hundreds or even thousands of antennas at the Base Station (BS), XL-MIMO offers extremely high spatial resolution, effectively transforming the array into a massive sensor capable of resolving fine-grained electromagnetic environments [2,3]. Furthermore, the continuous evolution of massive arrays—incorporating components like reconfigurable intelligent surfaces (RISs) and movable time-modulated arrays—has empowered novel capabilities beyond traditional data transmission, ranging from integrated sensing and communication (ISAC) [4] to covert and secure satellite-terrestrial networking [5,6].

Unlike traditional massive MIMO, XL-MIMO systems frequently operate in the radiative near-field region due to the significantly expanded Rayleigh distance. This introduces complex electromagnetic characteristics, most notably the spherical wavefront effect and spatial non-stationarity, which render traditional plane-wave assumptions invalid [7,8]. Recent channel modeling efforts emphasize the necessity of using 3D non-stationary models with visibility regions to accurately capture these near-field dynamics [9], which also fundamentally impacts multiple access strategies [10]. In this context, accurate Channel State Information (CSI) acquisition is not merely a communication prerequisite but a complex channel sensing task. The BS requires precise downlink CSI to design precoding matrices. However, the dimension of the channel matrix grows linearly with the number of antennas, leading to a “sensing data deluge” that exceeds the capacity of limited feedback control channels [11,12]. While Compressive Sensing (CS) techniques have been employed to reduce this sensing overhead, they often rely on strict sparsity assumptions that may not hold in complex near-field environments where scattering clusters are non-uniformly distributed. In recent years, Deep Learning (DL) has revolutionized the physical layer of wireless communications, offering a new paradigm for sensing data compression [13]. Seminal works, such as CsiNet [14], utilized autoencoders to compress channel matrices. Subsequent studies integrated attention mechanisms and multi-resolution architectures to further enhance reconstruction accuracy [15,16]. Building on these foundations, recent literature has rapidly expanded to address specific near-field challenges. For instance, lightweight autoencoders have been developed to alleviate feedback overhead in FDD systems [17], while advanced machine learning models have been proposed specifically for near-field CSI feedback and channel estimation [18,19]. Moreover, emerging AI-native paradigms, including Generative AI, have demonstrated tremendous potential in predicting CSI amidst severe spatial non-stationarity [20,21]. Despite these advancements, directly applying existing DL-based CSI feedback schemes to 6G XL-MIMO reveals two primary limitations:

- Neglect of Near-Field Sensing Sparsity: Most existing networks treat the channel matrix as a generic image, ignoring the specific Angle-Delay Domain sparsity caused by the limited scattering clusters in XL-MIMO environments. This leads to the inefficient allocation of neural network resources to noise rather than significant sensing features [22].
- Inefficiency of the Reconstruct-then-Precode Paradigm: Conventional approaches aim to minimize the Mean Squared Error (MSE) of the reconstructed channel. However, the ultimate objective of the sensing process in FDD systems is to maximize beamforming gain (spectral efficiency), not merely to reconstruct the raw data. Reconstructing the full high-dimensional channel at the BS before calculating the precoding matrix (e.g., via Singular Value Decomposition, SVD) is computationally expensive and introduces unnecessary latency [23]. Although recent deep neural network-based strategies have made strides in low-overhead beam management [24], integrating these into a true end-to-end precoding paradigm remains inefficient.

To address these challenges, this paper proposes an efficient, end-to-end limited feedback beamforming solution specifically tailored for the unique sensing characteristics of XL-MIMO. We argue that by synergizing physical domain knowledge (sparsity) with data-driven deep learning, high-performance beamforming can be achieved with significantly reduced sensing overhead.

The main contributions of this paper are summarized as follows:

(1) Analysis of XL-MIMO Spatial-Frequency Sensing Sparsity: We systematically analyze the energy distribution of XL-MIMO channels using hybrid channel models (COST 2100 and QuaDRiGa). Empirical analysis verifies that the channel energy is highly concentrated in specific regions of the Angle-Delay domain, motivating a physics-driven sensing compression strategy.
(2) Design of Spatial–Frequency Concentration Block (SFCB): Instead of processing the full raw CSI, we introduce the SFCB, a pre-processing module that acts as a “hard attention” mechanism. It dynamically screens features based on energy gradients, achieving efficient dimensionality reduction at the source (UE side) and significantly reducing the input size for the subsequent neural network.
(3) Development of Direct Integrated Precoding Network (DIP-I): We propose a lightweight end-to-end network, DIP-I, which maps compressed features directly to the precoding matrix. This design bypasses the explicit channel reconstruction stage, avoiding error accumulation and reducing the computational complexity of SVD operations at the BS.
(4) Validation in Realistic Scenarios: We evaluate the proposed scheme under complex indoor (COST 2100) and outdoor non-line-of-sight (NLOS) (QuaDRiGa) scenarios with a 512-antenna array. Results confirm that our approach outperforms separated feedback-precoding schemes in terms of effective sum-rate and computational efficiency.

Such high spatial resolution and near-field sensing capabilities enable diverse real-world applications. For instance, in smart factory environments, XL-MIMO can provide centimeter-level localization and high-throughput connectivity for industrial robots. In dense urban hotspots like stadiums or transit hubs, the proposed beamforming scheme can effectively mitigate interference through ultra-fine spatial multiplexing, ensuring robust service for thousands of concurrent users.

The remainder of this paper is organized as follows. Section 2 describes the XL-MIMO system model and channel characteristics. Section 3 details the proposed SFCB mechanism. Section 4 presents the DIP-I neural network architecture. Section 5 discusses the simulation results, and Section 6 concludes the paper.

## 2. System Model and XL-MIMO Channel Characteristics

### 2.1. XL-MIMO System Model

We consider a downlink XL-MIMO system where the Base Station (BS) is equipped with an extremely large uniform linear array (ULA) composed of $N_{t}$ antenna elements, serving a single-antenna user [25]. Table 1 presents the Mathematical Notations and Definitions.

#### 2.1.1. Array Layout and Field Partitioning

Assume the ULA antenna spacing is d, typically set to $d=\frac{\lambda }{2}$ (λ is the carrier wavelength). The physical aperture D of XL-MIMO is much larger than that of traditional arrays. Based on the distance r between the user and the BS, the spatial propagation environment is strictly divided into the far-field region and the near-field region, with the boundary defined by the Rayleigh Distance ($Z_{Rayleigh}$):

(1) $$Z_{Rayleigh}=\frac{2D^{2}}{\lambda }$$

In XL-MIMO scenarios, $Z_{Rayleigh}$ increases significantly (e.g., reaching several hundred meters for a 1024-antenna array at 30 GHz). When the user is located in the near-field region ($r<Z_{Rayleigh}$), the electromagnetic wavefront exhibits spherical curvature, rendering the traditional Plane Wave Model (PWM) inapplicable. Consequently, the Spherical Wave Model (SWM) must be adopted to accurately describe phase variations [26].

#### 2.1.2. Signal Transmission Model

In an Orthogonal Frequency Division Multiplexing (OFDM) system containing $N_{c}$ subbands, the received signal $y_{n}$ for the n-th subband is expressed as:

(2) $$y_{n}=h_{n}^{H}v_{n}s_{n}+z_{n}$$

where $h_{n}\in C^{N_{t}\times 1}$ is the downlink channel vector for the n-th subband; $v_{n}\in C^{N_{t}\times 1}$ is the precoding vector designed by the BS; $s_{n}$ is the transmitted symbol satisfying the power constraint $E\left[{\left|s_{n}\right|}^{2}\right]=1$; and $z_{n}∼CN\left(0,\sigma^{2}\right)$ denotes additive white Gaussian noise. The objective of this paper is to optimize $v_{n}$ under limited feedback constraints to maximize system spectral efficiency.

### 2.2. Multi-Scenario Channel Modeling

To verify algorithm robustness, this paper adopts complementary channel modeling methods:

(1) Indoor Scenario (COST 2100): For the 5.3 GHz band, the geometry-based stochastic COST 2100 model is adopted [27,28]. This model effectively captures the spatial non-stationarity caused by the large aperture of XL-MIMO arrays, where different antenna subsets may observe different scattering clusters.
(2) Outdoor Scenario (QuaDRiGa): For the 2.1 GHz outdoor NLOS scenario, the QuaDRiGa platform complying with the 3GPP TS 38.901 standard is utilized [29]. This platform accurately simulates SWM propagation characteristics and Visibility Region (VR) effects. The simulation area covers a specific range with the BS configured with 512 antennas.

### 2.3. Limited Feedback Architecture

**Figure 1:**
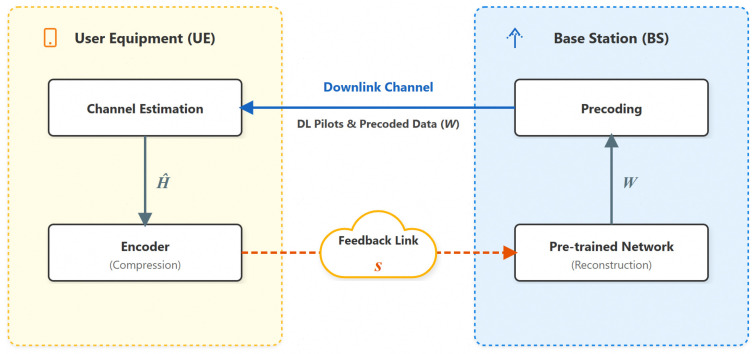
Limited feedback architecture diagram.

The complete limited feedback link consists of three stages (as shown in Figure 1):

(1) Channel Estimation: Downlink channel estimation at the UE side to obtain the downlink CSI matrix $\hat{H}$.

(2) Compressed Feedback: The UE utilizes the proposed dimensionality reduction mechanism (SFCB) and an encoder to map the CSI into a low-dimensional codeword s.

(3) Reconstruction and Beamforming: The BS side uses a pre-trained network to reconstruct the precoding matrix directly W from the feedback codeword.

## 3. Dimensionality Reduction Pre-Processing Mechanism Based on SFCB

### 3.1. Data Distribution Characteristics in Angle-Delay Domain

To solve the curse of dimensionality, it is first necessary to analyze the intrinsic distribution of the signal. A 2D Discrete Fourier Transform (2D-DFT) is used to map the spatial-frequency domain CSI matrix H to the Angle-Delay Domain matrix $\hat{H}$:

(3) $$\hat{H}=F_{d}HF_{a}^{H}$$

where $F_{d}$ and $F_{a}$ represent the DFT matrices for the delay (subband) and angle (antenna) dimensions, respectively [30,31].

**Figure 2:**
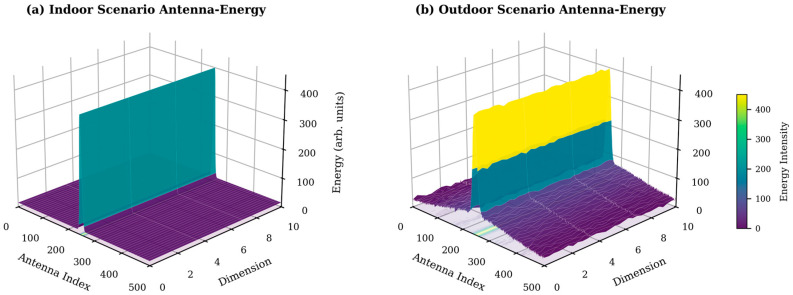
Energy distribution in the antenna dimension.

**Figure 3:**
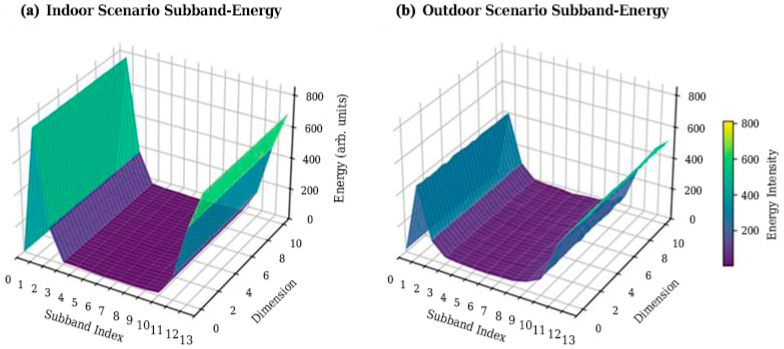
Energy distribution in the sub-band dimension.

For each data entry, the energy distribution proportions in both sub-band and antenna dimensions are statistically analyzed, and the results from multiple data samples are accumulated (as shown in Figure 2 and Figure 3).

It can be observed from the figures that the XL-MIMO channel exhibits significant Non-uniform Concentration in the Angle-Delay Domain: energy is not diffusely distributed but highly concentrated in a few “power centroid” regions. This physical characteristic provides a theoretical basis for selective pruning based on energy gradients [32].

### 3.2. Design of Spatial–Frequency Concentration Block (SFCB)

Based on the aforementioned XL-MIMO data distribution characteristics, a pluggable CSI compression precoding module is designed, with the core component being the SFCB (Spatial–Frequency Concentration Block).

#### 3.2.1. Algorithm Workflow

The design goal of SFCB is to maximize the retention of core intrinsic features reflecting XL-MIMO physical characteristics while drastically reducing data dimensions. Mathematically, this serves as a discrete optimization pre-processing step that maximizes the retained channel energy subject to specific dimensionality constraints. For a single CSI sample $\hat{H}$, SFCB employs a dynamic energy-containment mechanism to adaptively determine the optimal pruning window without manual tuning. The specific process is detailed in Algorithm 1:
**Algorithm 1.** Adaptive Spatial-Frequency Concentration Block (SFCB)
Require: Angle-Delay CSI matrix *H^^^ ∈ ℂ^N^_s_^× N^_a_*, Energy concentration ratio *Γ*. Ensure: Concentrated feature matrix *H_out_ ∈ ℂ^M^_s_^× M^_a_*.1:Step 1: Joint-Domain Energy Mapping. Calculate power density *E = |H^^^|^2^*;2:Step 2: Marginal Energy Projection.3:     ***e****_s_ = ∑_j=1_^N^_a_ **E**(·, j), **e**_a_ = ∑_i=1_^N^_s_ **E**(i, ·)*;4:Step 3: Adaptive Aperture Determination.5:   for each dimension *d* ∈ {*s*, *a*} do6:     ***e****_d_^sort^ = sort_desc(**e**_d_)*;7:     Find minimal *M_d_* s.t. (∑*_k_*_=1_*^M^_d_* **e***_d_*^sort^(*k*))/(∑*_k_*_=1_*^N^_d_* **e***_d_*(*k*)) ≥ *Γ*;8:     Identify feature-rich index set *ℐ_d_* based on *M_d_*;9:   end for10:Step 4: Spatial Topology Alignment.11:   *ℐ_s_* = sort_asc(*ℐ_s_*), *ℐ_a_* = sort_asc(*ℐ_a_*);  *▷ Recover original physical structure*12:Step 5: Dimensionality Resynthesis.13:   Slice feature matrix *H_out_ = H^^^(ℐ_s_, ℐ_a_)*;14:return *H_out_*

Step 1: Joint-Domain Energy Mapping. To identify the power distribution across space and frequency, we first construct the energy distribution map $E\in R^{N\times M}$ by calculating the squared modulus of each element in $\hat{H}$:

(4) $$E\left(i,j\right)={\left|\hat{H}\left(i,j\right)\right|}^{2},\;\forall i\in \left\{1,\ldots ,N\right\},j\in \left\{1,\ldots ,M\right\}$$

Step 2: Marginal Energy Projection. To evaluate the contribution of individual dimensions, the energy matrix is aggregated along the row and column dimensions.

Row-wise (Sub-band) Mapping: Summing E along the antenna dimension yields the subband energy vector $e_{row}\in R^{N}$, reflecting the frequency-domain energy contribution.

Column-wise (Antenna) Mapping: Summing E along the subband dimension yields the antenna energy vector $e_{col}\in R^{M}$, reflecting the spatial-domain energy contribution.

Step 3: Adaptive CDF-based Feature Pruning. To precisely determine the optimal dimensions ($N_{s},N_{a}$) for varying channel conditions, we utilize a Cumulative Distribution Function (CDF) thresholding strategy. Let “sort” (*e*) be the vector *e* sorted in descending order. The target dimension N is adaptively determined by satisfying a predefined energy containment ratio *Γ* (e.g., *Γ* = 0.95):

(5) $$minN\;s.t.\;\frac{\sum_{k=1}^{N}{\left[\mathrm{sort}\left(e\right)\right]}_{k}}{\sum e}\geq \Gamma$$

By calculating this for both $e_{s}$ and $e_{a}$, the mechanism automatically shrinks the window in sparse (LoS) conditions and expands it in rich-scattering (NLOS) environments to preserve significant path components. The indices $I_{s}$ and $I_{a}$ corresponding to the $N_{s}\;$and $N_{a}$ largest energy values are then selected.

Step 4: Spatial Topology Alignment. To ensure the extracted features maintain the underlying spatial-frequency correlations, the selected indices are re-sorted in ascending order:

(6) $${\tilde{I}}_{s}=\mathrm{sort}\_\mathrm{asc}\left(I_{s}\right),\;{\tilde{I}}_{a}=\mathrm{sort}\_\mathrm{asc}\left(I_{a}\right)$$

This step preserves the physical topology of the channel, which is essential for downstream feature learning via Convolutional Neural Networks.

Step 5: Dimensionality Resynthesis. Finally, the dimensionality-reduced matrix $H_{out}$ is synthesized by extracting the elements at the intersection of the screened indices:

(7) $$H_{out}=\hat{H}\left({\tilde{I}}_{s},{\tilde{I}}_{a}\right)$$

#### 3.2.2. Module Advantages

**Figure 4:**
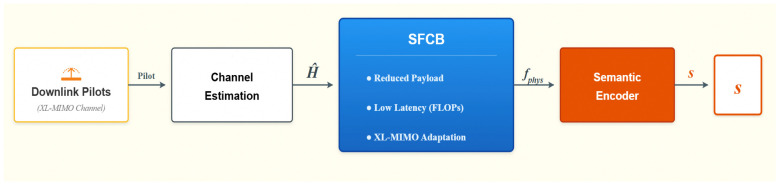
Schematic diagram of SFCB and feedback network.

SFCB adopts a Decoupled design, embedded as an independent pluggable module at the front end of the neural network on the UE side (as shown in Figure 4). Its advantages include:

(1) Autonomous Adaptation: The CDF-based thresholding eliminates the need for manual hyperparameter tuning for $\left(\eta_{freq},\eta_{ant}\right)$, allowing the system to handle non-stationary XL-MIMO channels across different UE locations and clusters.
(2) Reduced Load: Directly reduces the input dimensions and parameter count of the subsequent encoding network.
(3) Optimized Latency: Significantly lowers the Floating Point Operations (FLOPs) during the inference phase.

## 4. Proposed Two-Stage Beamforming Scheme Based on Dimensionality Reduction Precoding

### 4.1. Overall Architecture

**Figure 5:**
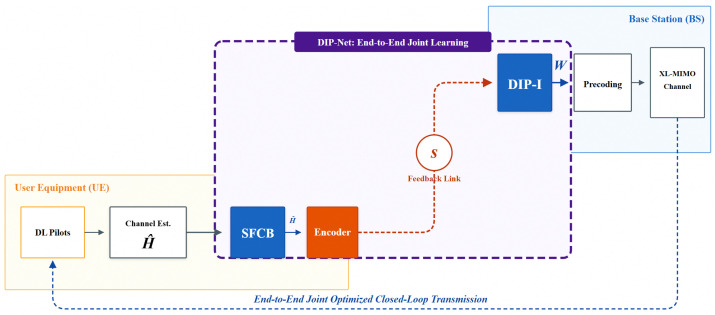
Design diagram of the limited feedback beamforming scheme.

This paper proposes a complete limited feedback beamforming scheme for XL-MIMO scenarios, named DIP-Net (as shown in Figure 5). The scheme decouples the processing flow into two stages: “Hard Compression” and “Soft Reconstruction”:

- Stage 1 (Hard Compression): SFCB performs physical-level feature dimensionality reduction, acting as a hard attention mechanism that forces the network to focus on the main path components where channel energy is concentrated.
- Stage 2 (Soft Reconstruction): The deep neural network DIP-I performs end-to-end feature extraction and precoding matrix generation.

### 4.2. Stage 1: Dimensionality Reduction Precoding

By introducing a compression ratio, the dynamic masking mechanism of SFCB outputs the feature matrix $H_{sub}$. This mechanism acts as a hard attention module, ensuring the network prioritizes significant channel components.

### 4.3. Stage 2: DIP-I Network Design

This section proposes the DIP-I (Direct Integrated Precoding Integrated Scheme). Unlike traditional CSI feedback (which first recovers H and then computes V), DIP-I aims to directly regress the optimal precoding matrix from the compressed codeword [29].

#### 4.3.1. Mathematical Description of System Flow

First, the raw CSI H undergoes SFCB dimensionality reduction to obtain $H_{sub}$. Subsequently, the encoder $f_{en}$ maps it to a codeword named c:

(8) $$c=f_{en}\left(H_{sub},\Theta_{en}\right)$$

After quantization and feedback, the decoder $f_{de}$ at the BS side directly generates the precoding matrix W:

(9) $$W=f_{de}\left(c,\Theta_{de}\right)$$

System performance is evaluated via the sum rate:

(10) $$R=\sum_{n=1}^{N_{c}}{log}_{2}\left(1+\frac{{\left|HW\right|}^{2}}{\sigma^{2}}\right)$$

In the data transmission process, $\sigma^{2}$ denotes the system noise power.

#### 4.3.2. DIP-I Network Architecture

**Figure 6:**
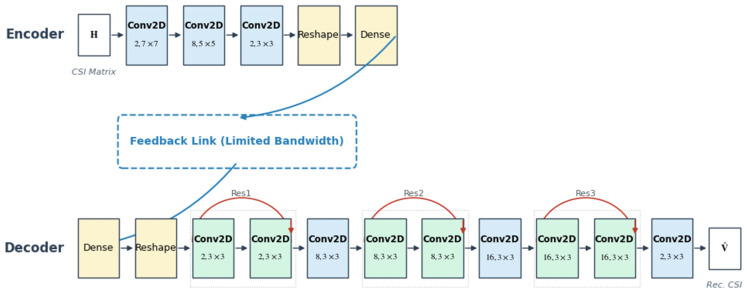
DIP-I limited feedback beamforming network structure.

DIP-I adopts a supervised learning strategy, with the network structure shown in Figure 6:

- Training Labels: Singular Value Decomposition (SVD) is performed on the unpruned perfect CSI to extract the principal eigenvector $v_{opt}$ as the ideal label.
- Encoder (UE side): The input is the data pruned by SFCB. The structure includes 3 convolutional layers (Conv2D, kernel counts 2-8-2) and 1 fully connected layer, responsible for feature extraction and codeword compression.
- Decoder (BS side): First recovers dimensions through a fully connected layer, followed by 3 cascaded Residual Blocks for deep feature reconstruction. The convolutional layers are configured with kernel counts 2-8-16, and the receptive field size is 3×3. Finally, a convolutional layer with 2 kernels and a receptive field of 3×3 outputs the precoding matrix. The residual block design effectively alleviates the gradient vanishing problem and enhances the learning capability for high-dimensional non-linear mapping [30].

#### 4.3.3. Optimization Objective and Training Procedure

To address the end-to-end precoding task, the training procedure is mathematically formulated as an optimization problem aimed at minimizing the discrepancy between the network’s predicted precoding matrix and the optimal SVD-derived matrix. Let $\Theta =\left\{\Theta_{enc},\Theta_{dec}\right\}$ represent the learnable parameters of the entire DIP-I network.

(1) Loss Function: The network parameters are optimized by minimizing the Mean Squared Error (MSE) loss function, which quantifies the Euclidean distance between the predicted precoding vector $V_{pre}$ and the ideal label $V_{opt}$. For a training batch of size *B*, the objective function is defined as:

(11) $$L\left(\Theta \right)=\frac{1}{B}\sum_{i=1}^{B}\left|\;\right|V_{opt}^{\left(i\right)}-f_{dec}\left(f_{enc}\left({\tilde{H}}_{a}^{\left(i\right)},\Theta_{enc}\right),\Theta_{dec}\right){\left|\;\right|}_{2}^{2}$$

(2) Optimization Algorithm and Hyperparameters: The optimization procedure employs the Adaptive Moment Estimation (Adam) optimizer, chosen for its robust convergence properties in non-convex neural network optimization. The specific training procedures are implemented as follows:

- Weight Initialization: Network weights are initialized using the Xavier (Glorot) normal distribution to maintain variance consistency across convolutional layers and prevent early-stage gradient explosion.
- Learning Rate Scheduling: The initial learning rate is set to $\eta =1\times 10^{-3}$. A dynamic learning rate decay strategy (e.g., ReduceLROnPlateau) is applied during the optimization process. If the validation loss fails to decrease for a consecutive number of epochs, the learning rate is scaled down by a factor of 0.5, ensuring fine-grained parameter updates near the global minimum.
- Batch Training: The dataset is divided into mini-batches (e.g., B=128). In each iteration, stochastic gradients $\nabla_{\Theta }L$ are computed through backpropagation, and the parameters Θ are iteratively updated until early stopping criteria are met or the maximum number of epochs is reached.

## 5. Simulation Results and Performance Evaluation

### 5.1. Simulation Parameter Settings

To comprehensively evaluate the performance of the proposed DIP-Net, an extensive dataset was constructed encompassing both indoor and outdoor communication scenarios. Experiments are based on the PyTorch (2.5.0) framework. Table 2 presents the key simulation and physical antenna parameters.

- Channel Models: COST 2100 [28] (5.3 GHz, Indoor)/QuaDRiGa (2.1 GHz, Outdoor NLOS).
- Antenna Configuration: BS with 512-antenna ULA with an antenna spacing of half a wavelength, and there are 13 sub-bands. In indoor and outdoor scenarios, place BS at the center of an area with a side length of 20 m and 40 m, respectively. Users are randomly distributed in the above areas.
- Dataset and Network Parameters: The dataset contains 120,000 training samples and 30,000 testing samples, containing 50% indoor scenarios and 50% outdoor scenarios. Epochs = 1000, Batch Size = 128 and Learning Rate = 0.001. The loss function is MSE, and the optimizer is Adam.
- Training Strategy: A two-step training method is adopted: during the training of the network, the SFCB module is added before the encoder to reduce the dimensionality of XL-MIMO data and uses the offline training mode. During the training, the neural network without the DIP-I module is first trained. The input of the network is the dimension-reduced data, and the supervision label is the precoding matrix obtained by performing SVD on the original CSI matrix that is not dimension-reduced. After the training is complete, the DIP-I module is added to quantize the feedback codewords, and then the decoder is trained for 500 epochs.

### 5.2. Performance Comparative Analysis

#### 5.2.1. Impact of SFCB on Overhead and Performance

**Figure 7:**
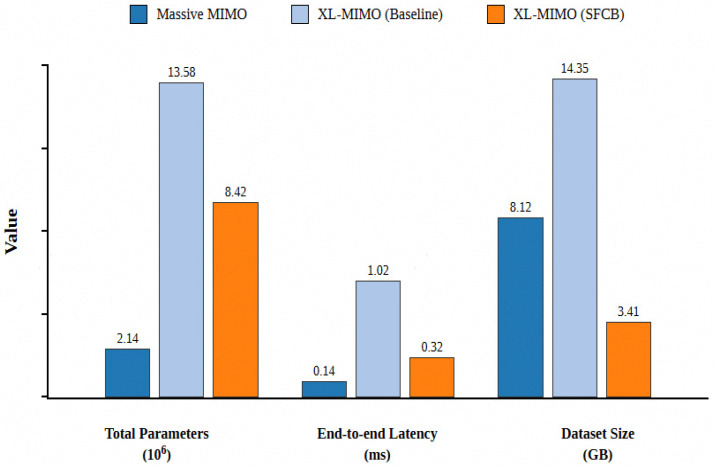
DIP-I neural network training parameters and inference latency under different datasets.

Figure 7 illustrates the comparison of network overhead before and after introducing SFCB. Taking 512 feedback codewords as an example, the introduction of SFCB significantly reduces input dimensions, resulting in a substantial decrease in the number of parameters and inference time for neural network training, as well as significantly reduced storage requirements.

**Figure 8:**
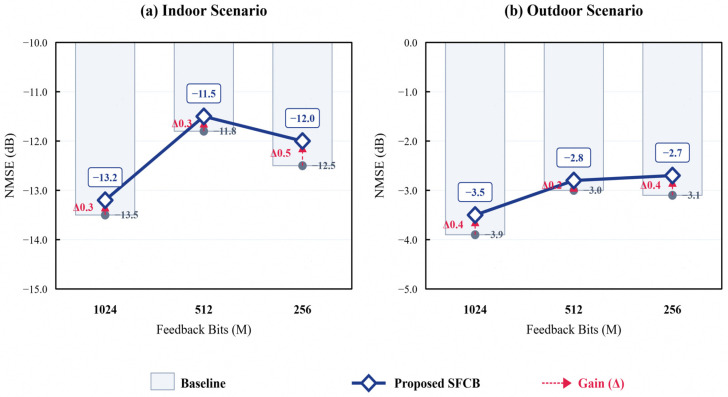
NMSE performance comparison of DIP-I neural network under different input modes.

To verify the impact of introducing the SFCB module on beamforming accuracy, this section compares the Normalized Mean Square Error (NMSE) performance of the network in indoor and outdoor scenarios when the input is SFCB-pruned data versus raw data, as shown in Figure 8.

Comparative experiments show that compared to inputting raw full-volume data, DIP-I with SFCB-pruned data input incurs only a minimal performance loss in NMSE (<0.5 dB). This confirms that SFCB can trade a negligible accuracy cost for significant computational efficiency gains, validating the effective utilization of XL-MIMO channel sparsity in the Angle-Delay Domain.

#### 5.2.2. Comparison of Limited Feedback Beamforming Schemes

To verify the architectural advantages of DIP-I, two schemes are compared:

- DIP-S (Separated Scheme): The network is responsible only for reconstructing CSI ($\hat{H}$), and the BS side calculates precoding via SVD.
- DIP-I (Integrated Scheme): The integrated scheme proposed in this paper. The network directly outputs the precoding matrix $\hat{V}$.

**Figure 9:**
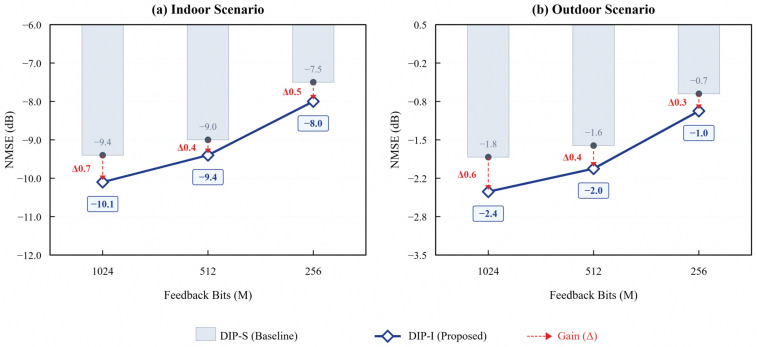
NMSE performance comparison of two different beamforming schemes.

Experimental results in Figure 9 show that the DIP-I scheme is significantly superior to DIP-S in precoding accuracy.

#### 5.2.3. Throughput Performance Analysis

Table 3 presents the average sum-rate performance under different feedback overheads and signal-to-noise ratios (SNRs). The experiment considers compression dimensions of 256, 512, and 1024, and introduces an ideal scheme (using perfect CSI for direct SVD) as the theoretical upper bound.

**Figure 10:**
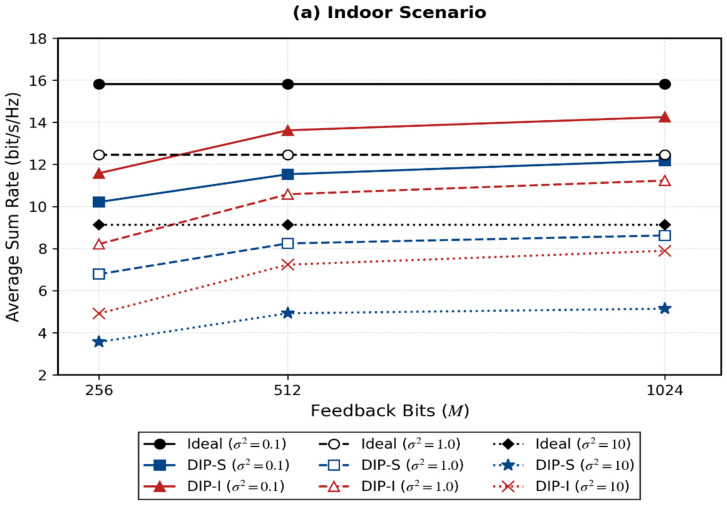

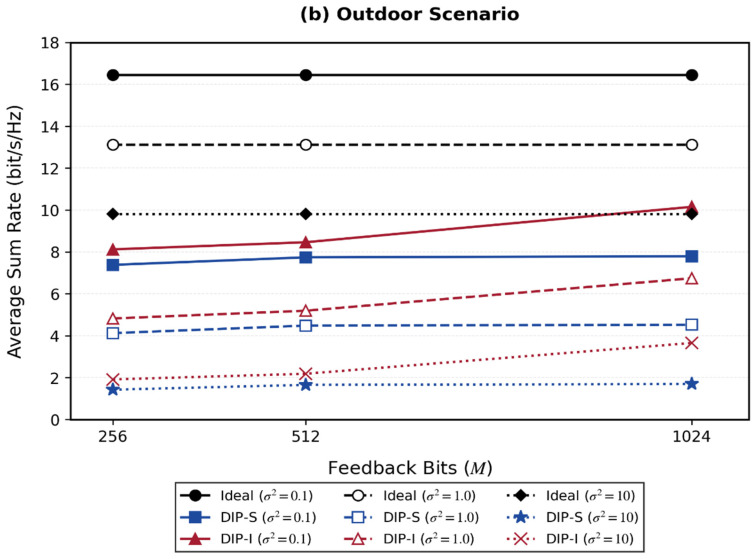
Comparison of average sum-rates of various schemes under different noise powers.

It can be seen that in the XL-MIMO scenario, the proposed DIP-I scheme outperforms DIP-S under different feedback overheads, and the average sum-rate is positively correlated with feedback accuracy. With larger indoor feedback overhead, the average sum-rate is very close to the theoretical optimal value. The comparison of average sum-rates for each scheme under different noise powers is shown in Figure 10.

The data indicate that in XL-MIMO scenarios, the proposed DIP-I scheme outperforms the DIP-S scheme under various feedback overheads and transmission noise conditions. Specifically, for the DIP-S scheme, since the channel CSI matrix must first be fed back to the BS side before performing precoding operations, the error between the CSI matrix received at the BS and the original CSI matrix leads to quality degradation of the precoding matrix during subsequent SVD operations, thereby affecting system performance. Furthermore, since this scheme requires further precoding of the received CSI matrix at the BS, it demands substantial additional computational resources and time, leading to high system complexity.

In contrast, compared to the two-step approach of DIP-S, the DIP-I scheme uses a neural network to directly output the precoding matrix and uses the matrix V obtained from perfect CSI precoding as the label. This integrates the feedback and beamforming processes, achieving better performance through global system optimization. The integrated method can better utilize feedback information and adjust it according to label information to generate superior precoding matrices. Additionally, the computational and time complexity of this scheme is lower than that of the step-by-step feedback beamforming scheme. Therefore, the DIP-I scheme proposed in this paper demonstrates superior performance in XL-MIMO scenarios.

To further evaluate the effectiveness of the proposed DIP-I scheme, we first provide a comprehensive comparison with existing representative literature in Table 4. Unlike traditional CSI feedback frameworks such as CsiNet [14] or Chen et al. [17], which are primarily designed for far-field stationary channels, our proposed method specifically addresses the unique electromagnetic characteristics of 6G XL-MIMO [33,34].

As illustrated in Table 3, the proposed DIP-I distinguishes itself in three key dimensions:

(1) Hybrid Domain Awareness: While prior works like Wu et al. [26] focus on near-field effects, our scheme simultaneously captures both spherical wavefront (near-field) and spatial non-stationarity, providing a more robust channel representation in realistic XL-MIMO deployments.
(2) Ultra-Low Feedback Overhead: By leveraging the physics-driven SFCB module to prune redundant spatial-frequency features before the encoding stage, our scheme achieves an ‘Ultra-Low’ feedback overhead, outperforming the compression efficiency of vanilla autoencoders.
(3) End-to-End (E2E) Efficiency: Unlike the conventional ‘reconstruct-then-precode’ paradigm seen in Refs. [8,26,28], DIP-I integrates feedback compression and precoding matrix generation into a single mapping process. This E2E design not only bypasses the accumulation of reconstruction errors but also significantly reduces the computational latency at the Base Station (BS), facilitating real-time beamforming in high-dimensional antenna systems.

## 6. Conclusions

This paper addressed the critical challenges of CSI sensing, feedback, and beamforming in 6G XL-MIMO systems. We proposed a synergistic architecture combining physics-based dimensionality reduction (SFCB) with end-to-end deep learning (DIP-I). The SFCB effectively exploits the angle-delay domain sparsity of near-field channels to reduce sensing processing overhead, while the DIP-I network learns a direct mapping to the optimal precoder, bypassing the latency-heavy reconstruction-SVD pipeline. Simulation results on COST 2100 and QuaDRiGa datasets confirm that our approach achieves state-of-the-art beamforming accuracy with significantly lower computational complexity. This solution is particularly promising for delay-sensitive 6G ISAC applications where hardware resources are constrained. Future Work and Trends: Integrating semantic communication with CSI feedback by incorporating Semantic Communications (SemCom) into the CSI feedback process and shifting from “reconstructing the original bits” to “conveying core semantic metrics” represents a significant direction for breaking through the Shannon limit and achieving ultra-low overhead feedback [35,36].

## Figures and Tables

**Table 1:** Mathematical Notations and Definitions.

Parameter Description	Symbol	Value
Number of BS antennas and OFDM subbands (sub-carriers)	*N_t_, N_c_*	Integer scalars
Antenna spacing of the ULA and carrier wavelength	*d, λ*	Physical scalars
Distance between UE and BS, and the Rayleigh Distance (boundary)	*r, r_Ray_*	Physical scalars
Received signal and additive white Gaussian noise for the *k*-th subband	*y_k_, n_k_*	Complex scalars
Downlink channel vector and precoding vector for the *k*-th subband	*h_k_, w_k_*	*ℂ* *^N^* *_t_* ^*× 1*^
Transmitted symbol and total power constraint (*𝔼[|s_k_|^2^] ≤ P*)	*s_k_, P*	Complex, Real scalar
Spatial-frequency CSI matrix and Angle-Delay domain CSI matrix	*H, H_AD_*	*ℂ* *^N^* *_t_* ^*× N*^ *_c_*
DFT matrices for antenna (angle) and subband (delay) dimensions	*F_t_, F_c_*	*ℂ* *^N^* *_t_* ^*× N*^ *_t_* *,* *ℂ* *^N^* *_c_* ^*× N*^ *_c_*
Neural network functions for CSI encoding and reconstruction	*f_enc_(·), f_dec_(·)*	Mapping functions
Learnable weights and biases for the encoder and decoder networks	*Θ_enc_, Θ_dec_*	Parameter sets
Continuous latent feature vector and quantized feedback bitstream	*z, s*	*ℝ^M^*, Binary vector
Quantization operator and its corresponding inverse (reconstruction)	*Q(·), Q^−1^(·)*	Operators
Reconstructed CSI matrix at the BS side	*Ĥ*	*ℂ* *^N^* *_t_* ^*× N*^ *_c_*
Objective loss function and the set of training channel samples	*ℒ* *,* *𝒟* *_train_*	Scalar, Dataset
Maximum number of training epochs and the current epoch index	*E_max_, t*	Integer scalars

**Table 2:** Key Simulation and Physical Antenna Parameters.

Parameter Description	Symbol	Value
Target Application Scenarios	-	Smart Factory/Dense Urban Hotspot
Antenna Array Type	-	Uniform Linear Array (ULA)
Number of BS Antennas	*M*	512
Carrier Frequency	*f_c_*	10 GHz
Antenna Spacing	*d* = 0.5 *λ*	1.5 cm
Total Array Aperture	*L*	7.665 m
Rayleigh Distance	*Z*	391.7 m
Near-Field Channel Model	-	Hybrid (COST 2100 & QuaDRiGa)
Optimizer	-	Adam (*η* = 10^−3^)
Signal-to-Noise Ratio	SNR	0–30 dB
Training Batch Size	*B*	128

**Table 3:** Comparison of average sum-rates for various schemes with noise power of 0.1 under different feedback overheads (bit/s/Hz).

Feedback *M*	Scheme	Indoor Scenario			Outdoor Scenario		
		*Q* = 2	*Q* = 3	*Q* = 4	*Q* = 2	*Q* = 3	*Q* = 4
256	Ideal	16.00	16.00	16.00	16.41	16.41	16.41
	**DIP-I (Proposed)**	**9.85**	**10.38**	**11.27**	**5.94**	**6.28**	**8.26**
	DIP-S (Baseline)	8.54	9.75	10.08	5.83	6.06	7.67
512	Ideal	16.00	16.00	16.00	16.41	16.41	16.41
	**DIP-I (Proposed)**	**10.15**	**11.12**	**13.68**	**6.27**	**7.08**	**8.47**
	DIP-S (Baseline)	9.07	10.28	10.87	6.05	6.67	7.39
1024	Ideal	16.00	16.00	16.00	16.41	16.41	16.41
	**DIP-I (Proposed)**	**10.98**	**12.07**	**14.69**	**6.36**	**7.18**	**9.87**
	DIP-S (Baseline)	9.68	10.79	10.97	6.24	6.79	7.46

**Table 4:** Comparison of Proposed Scheme with Existing Literature.

Reference	Array Scale (Number of Ant.)	Channel Model (Near-Field/Non-Stat.)	Core Methodology	Feedback Overhead	E2E Design
CsiNet [14]	[32, 64]	Far-field/Stationary	Vanilla Autoencoder	High	No
Wu et al. [10]	[256, 512]	Near-field/Stationary	LDMA/Beam-focusing	Moderate	No
Chen et al. [17]	[128, 256]	Far-field/Non-stat.	Lightweight AE	Low	No
Zhao et al. [24]	[512, 1024]	Near-field/Non-stat.	Beam Management DNN	Low	Yes
**Proposed** **(DIP-I)**	**[512, 1024]**	**Hybrid Near-field** **& Non-stationary**	**Physics-driven SFCB** **& Integrated Precoding**	**Ultra-Low**	**Yes**

## Data Availability

The original contributions presented in this study are included in the article. Further inquiries can be directed to the corresponding author.
